# Evaluating the Virucidal Activity of Disinfectants According to European Union Standards

**DOI:** 10.3390/v13040534

**Published:** 2021-03-24

**Authors:** Patryk Tarka, Aneta Nitsch-Osuch

**Affiliations:** Department of Social Medicine and Public Health, Medical University of Warsaw, Oczki Street 3, 02-007 Warsaw, Poland; anitsch@wum.edu.pl

**Keywords:** virucidal activity, suspension methods, carrier methods, coronavirus

## Abstract

The disinfection of surfaces in medical facilities is an important element of infection control, including the control of viral infections such as severe acute respiratory syndrome coronavirus 2 (SARS-CoV-2). Preparations used for surface disinfection are typically characterized via their activity against test organisms (i.e., viruses, bacteria and fungi) in the laboratory. Typically, these methods use a suspension of the test organism to assess the bactericidal, fungicidal or virucidal activity of a given preparation. However, such suspension methods do not fully imitate real-life conditions. To address this issue, carrier methods have been developed, in which microorganisms are applied to the surface of a carrier (e.g., stainless steel, glass and polyvinyl chloride (PVC)) and then dried. Such methods more accurately reflect the applications in real-life clinical practice. This article summarizes the available methods for assessing the virucidal activity of chemical disinfectants for use in medical facilities based on the current European standards, including the activity against coronaviruses.

## 1. Introduction

As a result of the severe acute respiratory syndrome coronavirus 2 (SARS-CoV-2) pandemic, the interest and demand for virucidal disinfectants have increased. Some studies have evaluated the virucidal activity of chemical preparations against coronaviruses [1,2,3]. However, viruses (including coronaviruses) can persist on surfaces for several days to several months [4,5,6,7,8]. Additionally, the susceptibility of viruses to chemical disinfectants varies depending on their structure. Therefore, these factors must be considered when assessing the virucidal activity of chemical disinfectants.

**Figure 1:**
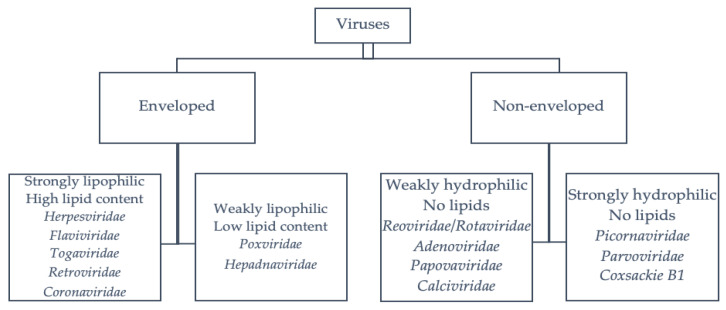
Classification of the viruses according to their structure [9,11].

Enveloped viruses are more susceptible to chemical disinfectants than nonenveloped viruses [9,10]. Nonenveloped viruses with strong hydrophilic properties (e.g., poliovirus, hepatitis A virus (HAV) and parvoviruses) are the most resistant to chemical disinfectants [9,10]. In contrast, those with reduced hydrophilic properties (e.g., adenoviruses, rotaviruses, noroviruses and caliciviruses, among others) are slightly more sensitive to chemical disinfectants [9,10]. Enveloped viruses with a low lipid content, including hepatitis B virus (HBV) and poxviruses, are also sensitive to disinfectants but are more resistant than enveloped viruses with a high lipid content [9,10]. The viruses that are the most sensitive to chemical disinfectants are enveloped viruses with a high lipid content, including coronaviruses, the hepatitis C virus (HCV), HIV and herpes viruses. A summary of the major viruses classified according to their structure and lipid content is shown in Figure 1.

A second important factor for disinfection is the virus environment. Viruses present in suspension are more easily inactivated by chemical agents than those in a dried form on surfaces [12,13,14]. Moreover, those viruses dried on surfaces contain a high protein load from blood and saliva that may protect them from chemical disinfectants [13]. Therefore, when decontaminating surfaces and equipment, both the type of virus (enveloped or nonenveloped) and the virus environment must be considered.

Indeed, the Technical Committee 216 (TC 216) “Chemical disinfectants and antiseptics” of the European Committee for Standardization (CEN) has been developing methods for testing the efficacy of disinfectants in Europe since 1989 [15]. The TC 216 has introduced a three-phase model for testing chemical disinfectants and antiseptics [16], as summarized below:

- Phase 1 (suspension) tests are performed to determine whether a chemical disinfectant or antiseptic has bactericidal, fungicidal, yeasticidal or sporicidal activity without regard for the specific areas of application. Phase 1 tests cannot be used for any product claim.
- Phase 2/Step 1 tests use quantitative suspension methods, in which the organisms are exposed to the chemical disinfectants or antiseptics at various concentrations, times and temperatures and with the addition of interfering substances. These tests confirm the performance of the product under laboratory conditions similar to the intended use (e.g., on instruments or surfaces in the medical area). An example of a Phase 2/Step 1 standard is the EN 14476:2013 virucidal activity standard [17].
- Phase 2/Step 2 tests are based on carrier methods under conditions simulating a practical use. In the medical area, these include standards for disinfecting instruments, such as EN 14561:2008 for assessing the bactericidal activity [18], EN 14562:2006 for assessing the fungicidal and yeasticidal activity [19], EN 14563:2008 for assessing the mycobactericidal and tuberculocidal activity [20] and EN 17111:2018 for assessing the virucidal activity [21]. For surface disinfection, two types of actions are distinguished: those without a mechanical factor (i.e., the draft FPREN 17387 for bactericidal, yeasticidal and fungicidal activity [22] and the EN 16777:2018 for virucidal activity [23]) and those involving a mechanical factor (i.e., EN 16615:2015 for bactericidal and yeasticidal activity [24]).
- Phase 3 tests were intended to be conducted under practical in-use conditions, but there are currently no draft or standards.

The current standards for assessing the virucidal activity of chemical disinfectants are summarized in Table 1.

## 2. Suspension Methods for Testing Virucidal Activity

The TC 216 has developed the EN 14476:2013+A2:2019 standard on quantitative suspension tests for evaluating virucidal activity in the medical area (Phase 2/Step 1) [10,17]. The standard distinguishes four groups of products: (1) those intended for use as hygienic hand rubs and handwashes; (2) preparations for disinfecting instruments; (3) surface disinfectants and (4) preparations for disinfecting textiles.

For products intended for hygienic hand disinfection and disinfecting surfaces, three ranges of activity have been introduced: virucidal activity (i.e., full virucidal activity), limited virucidal activity and virucidal activity against enveloped viruses. As a criterion for virucidal activity, the standard assumes a reduction in the infectious titer of at least 4-log 10 (the difference between the infectious titer of the virus in the control mixture and the infectious titer of the virus in the test mixture, containing a specific concentration of the test product). A 4-log 10 drop in infectious virus titer equates to a loss of infectivity of 99.99%. The disadvantage of this method is that the viruses are exposed to a large amount of the disinfectant in suspension, which makes them easier to inactivate.

Moreover, in the German methodology for testing the "full virucidal activity" of disinfectants, the nonenveloped Simian virus 40 (SV40), belonging to the polyomaviruses, was introduced [26,27]. Both the limited and full virucidal activity were assessed by suspension and carrier tests. Simian virus 40 (SV40) is used as a surrogate for the human papillomavirus (HPV).

HPV is resistant to certain high-level disinfectants (HLD), such as glutaraldehyde and ortho-phthalaldehyde, in both suspension [28] and carrier tests [29].

## 3. Suspension Tests Versus Carrier Methods for Evaluating Virucidal Activity

Recommendations on the practical applications of the virucidal agents can be drawn from the results of the suspension tests only to a limited extent, as the conditions found in homogeneous suspensions are rare in practice [24]. Moreover, viruses in suspension are much easier to inactivate than viruses present on surfaces [13]. In comparison, when using carrier methods, the testing system is complicated by introducing the carrier. In such tests, microorganisms are applied to the surface of the carrier (made of various materials, e.g., stainless steel, glass, plastic and fabrics) and then dried, which more accurately imitates the practical conditions. As such, the concentration and action times are usually much greater than those obtained with the suspension methods. Unfortunately, there were no European guidelines for carrier tests until May 2012, when the German Association for the Control of Viral Disease (DVV) published new recommendations for testing virucidal activity on nonporous surfaces in Germany [30].

The selection of an appropriate model virus is crucial for carrier testing. Such model viruses must be highly resistant to chemical disinfectants and drying and achieve a high titer in the culture [13]. Indeed, the poliovirus was excluded, because it is sensitive to the drying process: the loss of the infectious titer of the poliovirus during drying is approximately 3-log 10 [10]. When evaluating enveloped viruses using the carrier method, the modified vaccinia virus Ankara (MVA) virus was chosen as the test virus, as it is considered to be the most resistant enveloped virus [31]. MVA is also safer for medical personnel than the vaccinia virus Lister Elstree (VACV) used in the suspension method, and its destruction guarantees the inactivation of viruses such as HIV, HBV, HCV, coronaviruses or filoviruses (including the Marburg and Ebola viruses) [10,31].

For evaluating nonenveloped viruses using the carrier method, parvoviruses (with a very high resistance to both heat and chemicals) are ideal test organisms. According to the EN 14476:2013 +A2:2019 standard [17], the bovine parvovirus (BPV) is a suitable test virus for assessing the chemical and thermal processes, because it is highly stable and resistant to high temperatures. However, as a single-stranded DNA virus, BPV requires special conditions for proliferation [13]. Therefore, the mouse parvovirus Minute Virus of Mice (MVM), which is easier to cultivate, was proposed as a model virus for use in carrier tests [13]. The adenovirus type 5 (AdV-5), a clinically relevant virus, can also be used as an appropriate model for carrier testing [13].

## 4. Carrier Methods for Testing Virucidal Activity According to European Union Standards

### 4.1. Surface Disinfection without Mechanical Action

The test method and requirements for evaluating the virucidal action of chemical disinfectants without mechanical action for use in the medical field on nonporous surfaces were published in early 2019 [21]. When assessing the virucidal activity of preparations intended for surface disinfection, the complete virucidal activity can be evaluated using the poliovirus (i.e., using the suspension method as per the EN 14476:2013+A2:2019 standard [17]), with the adenovirus and murine norovirus (MNV); a limited spectrum virucidal activity can be assessed using the adenovirus and MNV. For virucidal activity against enveloped viruses, VACV and MVA are used.

### 4.2. Surface Disinfection with Mechanical Action

Most cleaning and surface disinfection processes in medical facilities are carried out using the wiping method (i.e., a mechanical factor). The European standard EN 16615:2015 describes a mechanical process of rubbing in four fields, starting with the contaminated field 1, then fields 2–4, and then back to the starting field (termed the four-field test) [24]. However, this method only describes the disinfection and transmission of vegetative forms of bacteria and yeasts using disinfectant wipes, without testing the virucidal activity [24]. Since this standard was published, the sporicidal activity of disinfectant wipes against *Clostridium difficile* [32]—as well as the yeasticidal activity against *Candida auris* [33] and virucidal activity against MNV (as a surrogate for human noroviruses), AdV-5 and SV40 [34]—have been assessed using the four-field test. Indeed, Becker et al. showed that the four-field test can accurately evaluate the virucidal activity of disinfectant wipes. The wipes were examined for contamination with viral materials. On the per acetic-based wipe, no residual virus was detected after usage. On the wipes based on 2-propanol and quaternary ammonium compounds (QACs), viruses were detectable. It proved that the viruses were not inactivated but transferred onto the wipes material [34]. As a result, a working group has begun developing work item WI00216104, a virucidal activity standard for testing surface disinfection using a mechanical factor [35].

### 4.3. Assessing Airborne Room Disinfection by an Automated Process

Conventional disinfection methods are limited by relying on the operator to ensure proper surface selection, product preparation and disinfectant contact time. “No-touch” automated room disinfection systems reduce the reliance on operators and can, therefore, improve the effectiveness of the final disinfection [36]. Suspension tests for chemical disinfectants are inappropriate for evaluating airborne room disinfections by an automated process [37]. Such systems could previously only be tested based on the national French standard NF T 72-281 (2014) [38]. However, in April 2020, the European Committee for Standardization published the EN 17272:2020 standard for evaluating airborne room disinfection by an automated process (including the determination of bactericidal, mycobactericidal, sporicidal, fungicidal, yeasticidal, virucidal and phagocidal activities) [39]. The recommended test viruses for use in the medical field are the adenovirus and MNV [39].

## 5. Assessing Instrument Disinfection

To assess the utility of chemical disinfectants for instruments, the following carrier method standards were developed: EN 14561:2006 for bactericidal activity [13], EN 14562:2006 for fungicidal and yeasticidal activity [19], EN 14563:2008 for mycobactericidal or tuberculocidal activity [15] and EN 17111:2018 for virucidal activity [21]. The carriers used for these tests are prepared on glass plates. Depending on the intended use of the preparation, different strains of the viruses are used [21]. For example, the MVA virus or VACV (strain Elstree) is used in the carrier method for evaluating pre-disinfection preparations [21]. Preparations for pre-disinfection do not have to cover a broad spectrum of virucidal activity, as their use is aimed at inactivating enveloped viruses transmitted through the blood and preventing infections among the personnel preparing the instruments for sterilization. To claim virucidal activity, the product must pass standard EN 14476 with the poliovirus, adenovirus and MNV and with the adenovirus and MNV in the carrier method [21].

In contrast, preparations are required to have a wide range of virucidal activity, against both enveloped and nonenveloped viruses, inter alia, for disinfecting medical devices (e.g., flexible endoscopes), where sterilization does not follow the disinfection process. In the case of chemical–thermal disinfection in a machine process, the test virus is changed from the bovine parvovirus to the murine parvovirus, which is easier to cultivate [13].

Becker et al. [40] recently assessed the virucidal activity of peracetic acid for instrumental disinfection according to the EN 17111 standard [21]. The preparation based on peracetic acid was effective according to the EN 14476 standard and, also, showed effectiveness against the adenovirus and MNV in the carrier test; however, a higher concentration of peracetic acid was required in the carrier test compared to the suspension test method with MNV [40]. The findings by Becker et al. support the previous claims that suspension tests may not accurately reflect real-life conditions and may overestimate the activity of chemical disinfectants. Additionally, in the carrier tests, MVM showed a greater stability than in suspension tests against glutaraldehyde [41].

## 6. Evaluating Hand Disinfection

Hands are the most common vector for transmitting infections in medical facilities. Currently, the virucidal activity of preparations intended for hygienic hand disinfection is tested by the suspension method, according to the EN 14476:2013+A2:2019 standard [17]. The standard specifies the minimum range of test viruses. The viruses used for full virucidal activity testing include the poliovirus, adenovirus and MNV; those for limited virucidal activity include the adenovirus and MNV and VACV is used for virucidal activity against enveloped viruses.

Some viruses, such as HAV, show a high resistance to ethanol [42], which is considered to be the most virucidal of all alcohols used in hand disinfection products [43]. Thus, as the tests with suspension methods do not fully reflect the conditions found in real-life practice, the Technical Committee 216 (TC 216) for Disinfection and Antisepsis of the European Committee for Standardization presented the draft standard prEN 17430 [44]. This standard describes the requirements and methods for assessing the virucidal activity of hygienic hand rubs using MNV as the test virus [44]. Using the methodology described in prEN 17430, Eggers et al. assessed the virucidal activity of three preparations for hand disinfection (based on ethanol in a gel 86% *v/v*, ethanol 89.5% *v/v* and ethanol 72.4% *v/v*) with 18 volunteers [45]. They found that the three preparations showed significant reduction factors (RF) against MNV compared to the reference solution (i.e., RFs of 1.96 ± 0.64, 2.49 ± 0.59 and 2.61 ± 0.50, respectively) [45]. Thus, all three hand rubs passed the virucidal efficacy criteria stated in the draft prEN 17430.

## 7. Conclusions

Preparations in which the manufacturer declares the virucidal activity should be tested using both suspension and carrier methods. In the case of SARS-CoV-2, disinfectants that meet the suspension test standard EN 14476+A2:2019-08 (for hands, surfaces and instruments); the carrier test standard EN 16777:2019-01 (for surfaces) and the carrier test standard EN 17111:2018 (for instruments), as well as the automated disinfection methods that meet the EN 17272:2020 standard, can be used for disinfection. The virucidal activity against enveloped viruses is suitable to inactivate the SARS-CoV-2 virus. Although no further testing with the SARS-CoV-2 virus is required, additional tests using viral surrogates, such as the murine coronavirus (MHV), bovine coronavirus (BCV), feline infectious peritonitis virus (FIPV) or human coronavirus 229 E (used in a biosafety level 2 laboratory), may provide useful supplemental information [46,47,48,49].

## Figures and Tables

**Table 1:** Summary of the standard tests for evaluating the virucidal activity of chemical disinfectants [25].

Application	Phase 2/Step 1 (Suspension Test) EN 14476:2013+A2:2019	Test Viruses	Phase 2/Step 2 (Carrier Test)	Type of Virucidal Activity Range	Test Viruses
Hygienic hand rub disinfection	Virucidal activity	Poliovirus Adenovirus Murine norovirus	prEN 17430	Virucidal activity (draft)	Murine norovirus
	Limited spectrum virucidal activity	Adenovirus Murine norovirus			
	Virucidal activity against enveloped viruses	Vaccinia virus			
Surface disinfection (without mechanical action)	Virucidal activity	Poliovirus Adenovirus Murine norovirus	EN 16777:2019-01E	Virucidal activity	Poliovirus (results from the suspension test standard EN 14476:2013+A2:2019) Adenovirus Murine norovirus
	Limited spectrum virucidal activity	Adenovirus murine Norovirus		Limited spectrum virucidal activity	Adenovirus Murine norovirus
	Virucidal activity against enveloped viruses	Vaccinia virus		Virucidal activity against enveloped viruses	Vaccinia virus
Airborne room disinfection by automated process	Suspension tests are unsuitable for this type of process, especially for gases		EN 17272:2020	Virucidal activity	Adenovirus Murine norovirus
Instrument disinfection temperature <40 °C	Virucidal activity	Poliovirus Adenovirus Murine norovirus	EN 17111:2018-12E	Virucidal activity	Poliovirus (results from the suspension test standard EN 14476:2013+A2:2019) Adenovirus murine norovirus
				Virucidal activity against enveloped viruses (products for pre-disinfection)	Vaccinia virus
Instrument disinfection temperature ≥40 °C	Virucidal activity	Murine parvovirus	EN 17111:2018-12E	Virucidal activity	Murine parvovirus
Chemical and thermal disinfection of textiles	Virucidal activity	Murine parvovirus	No standard		

## Data Availability

Not applicable.
